# Cases of Amputation in Private Practice

**Published:** 1846-07

**Authors:** William M. Boling

**Affiliations:** Montgomery, Alabama


### WESTERN JOURNAL

OF

### MEDICINE AND SURGERY.

JULY, 1846.

ART. I.—*Cases of Amputation in Private Practice.* By WILLIAM M. BOLING, M.D., of Montgomery, Alabama.

A comparison of the success attendant on any particular operation, or class of operations, in private practice, with that in hospitals, would, perhaps, not be entirely devoid of interest. One element for such comparisons can readily be procured from the numerous publications of cases, and tabular views by hospital surgeons and physicians; but as in private practice the number of operations of any particular kind, performed by any one operator, must generally be limited; no comparison can be instituted from the published cases of any one. By taking the aggregate of cases published by a number of individuals, such comparisons might be made.

It seems to be a common impression, that the result of operations is generally more successful in private, than in hospital practice; the advantages of the latter, arising from the greater dexterity of the operator, from frequent practice, being, it is said, more than balanced by the better air, &c., of private abodes.

In looking recently over the few amputations performed by myself, I was surprised at the great proportionate mortality, and for this reason only thought of offering them for publication; believing, however, that the average mortality of a larger number of cases, collected from private practice, would be much less.

Operations performed in hospitals are generally done by men in the daily habit of operating; conseqently the patient has all the benefit of that manual dexterity which is given by practice. This, however, is more limited than at first thought one would suppose, as the manner, in regard to neatness of execution, in which an operation is performed, and the time occupied in it, will, as a general rule, have very little influence over the ultimate result: not that operations are not occasionally performed so badly as to influence the result, but this is rarely the case. True, the practiced surgeon will perform an operation in a few seconds less of time, and may separate parts with the knife at two strokes, which would require three from one of less practice. He will do it too with apparent ease and facility, while to the other it appears a work of labor; still the issue perhaps is not influenced by this once in a hundred operations. But what is of infinitely more importance, as influencing the general result, is the knowledge acquired by practical experience, of the cases suitable for operations, under what circumstances an operation would be useless or hopeless, and the proper moment for its performance when decided upon.

Hospitals being always located in cities, consequently within the reach of many, nearly all the operations which are required for accidental injuries may be performed almost immediately after the infliction, and before constitutional disturbance from inflammation, gangrene, &c., can arise—a matter of no small importance in many instances. While in private practice, or rather the country portion of private practice, the patients frequently being at a distance, this cannot be accomplished. Hospital patients have generally the advantage of the constant presence of a physician, or well qualified student, in case of any unforeseen accident or emergency. They have also the benefit of experienced nurses, who know their duty, and are satisfied with performing it, and no more; while the patient in private practice has to contend with that pest of society in general, and of sick persons and physicians in particular, the would-be doctresses—the old women, (old men, in this respect, occasionally put on the petticoats,) with her all-curing poultices, plasters, 'dr*a*ps,' or clysters.

The circumstances which are supposed to favor the result of operations in private practice, are these: The patients are said generally to be of a class superior to those of hospitals; but superiority in rank or wealth does not necessarily imply superiority in morals or habits, and the advantage in such cases depends solely on the constitution of the patient—whether impaired or not by vicious and immoral habits—for the hospital patient, after he becomes such, has all the advantages of nursing, attendance, &c., procurable by the generality of patients in private practice.

There is probably but little difference in the constitutional peculiarities, as produced or modified by the habits or morals of the individuals themselves, of patients in private and in hospital practice. I speak here in relation to operations required on account of injuries received from external violence of any kind, and more especially with reference to southern practice. The ruffian or bully, who receives his stab or gunshot wound at an election brawl in the country, a subject for private practice, has a constitution perhaps as much impaired by excess and dissipation, as the subject for hospital practice, who receives his injuries at a city bagnio or drinking-shop.

If, in the south, the patient in private practice has the benefit of any constitutional superiority, it is in the case of the negro. The regular alternation of labor and rest, a due supply of wholesome nutriment, want of care and mental anxiety, and above all, an entire absence of those effects produced by excessive indulgence in intoxicating drinks, and dissipation generally, place him, in regard to constitution, far above the white laboring classes of the larger cities. In a practice of ten years in Alabama, I have neither seen nor heard of a case of delirium tremens in a slave. The greater liability of the negro in the south to tetanus, after injuries, admitting the supposition to be correct, may probably overbalance this advantage.

The great advantage to patients in private practice, of a purer air than that of hospitals, cannot be questioned.

The danger of the hospital patient is much increased by his exposure to contagious diseases, more especially erysipelas, which in some hospitals has, heretofore, if such is not even yet the case, added materially to the mortality arising from surgical operations. This is perhaps the only real and decided advantage of the patient in private practice, while the disadvantages under which he labors are numerous. I do not here take into consideration the *luxuries* of a sick room, but have reference merely to the necessaries calculated to influence the result. The former, of course, can only be procured in private practice, and among the wealthy.

Case I.—W. C., a stout, healthy farmer, about 35 years old, received in his left arm, on the 15th of July, 1837, a wound caused by the discharge of a gun containing 21 buckshot, and a rifle ball. The muzzle of the gun was within six feet of him at the time it was discharged. The load was received on the anterior surface of the forearm, about two inches below the elbow joint, shattering both bones very much, splitting the upper end of the ulna (as was afterwards discovered) as far up as the joint, and carrying several fragments with it, as it passed out from the posterior face of the arm. The muscles were almost entirely divided; the lower portion of the arm with the hand, hanging literally by two small strips of skin, one on the radial, and the other on the ulnar side of the arm. The hemorrhage was considerable.

On the 16th, I was called to see him, but as the messenger was unaware of the extent of the injury, and as the request was to come and *set* a broken bone, I went unprepared for amputating. The distance was eighteen miles, consequently I could not procure my instruments in time to operate before the following morning.

On the morning of the 17th I amputated the arm by the circular operation about two inches above the elbow joint. He recovered in a reasonable time without any unfavorable symptoms. The stump was a bad one in consequence of a redundancy of integuments.

Case II.—A negro man, 30 years of age, has had a large ulcer of many years' standing, causing swelling of the foot and leg from a short distance below the knee to twice the natural size. It causes him much pain and constitutional disturbance, and seems incurable, as numerous and continued efforts have been made for that purpose.

He was a patient of Dr. Holt, at whose request, January 30th, 1841, I amputated the leg a short distance below the knee, by a modification of the circular operation, leaving the skin and mass of muscles forming the calf of the leg considerably longer than the integuments anteriorly.

According to the method of Liston, we did not attempt to dress the stump for several hours after the operation, and in doing so found the sensibility of the parts augmented to such a painful degree, that we had to desist till the patient could be brought under the influence of a large dose of tincture of opium. The slightest effort to lay the parts in apposition, caused violent spasmodic action in the muscles, in consequence of which every arterial branch was caused to throw out blood profusely, and he lost more during the few minutes we were making the attempt, than he did during the operation. This one experiment of the kind satisfied me.

The integuments were rather short, and the stump healed slowly.

Case III.—A negro girl, about 11 years old, received in the bottom of her foot a puncture with a nail. By neglect and mismanagement the foot was caused to mortify. The principal part of the mischief was probably done by dipping it in concentrated ley, such as is used in families for making soap.

Probably about three weeks after the injury, Dr. Ames was called to see her, and at his request I visited her with him a day or two after, for the purpose of amputating the limb. The whole foot was then destroyed; the bones protruding at various points, and mixed with the sloughing flesh, constituted an indiscriminate mass of fœtid rottenness. Her emaciation was extreme, and there were several abscesses on various parts of her body, (the consequence of purulent absorption), from which pus was profusely discharged. Her pulse was small, quick, and corded; skin hot and dry; countenance anxious and haggard. So extreme was her debility, that it was a question between Dr. Ames and myself whether she could sustain the loss of blood necessarily attendant on the operation, husband it as we might.

July 6th, 1841. The amputation was performed some distance below the knee, and notwithstanding every precaution, the loss of blood was much more considerable than we had anticipated. Perfectly thin, and almost colorless, it oozed from every point of the cut surface. By the use of stimulants, however, she sustained the operation, notwithstanding the loss of blood. It was my intention to have made a flap posteriorly, but there was nothing of which to form it, the small mass of muscle, not larger than a man's finger, merely filling the space behind, between the tibia and fibula. The circular operation was performed.

Contrary to all expectation she recovered. The principal remedies employed by Dr. Ames in her treatment, were I believe, bark, wine, and rich soups.

Case IV.—January 8th, 1842. A negro boy, about eight years old, while gathering chips near where another was chopping wood, placed his hand in the way of the axe as it descended, by which it was nearly severed from the arm, obliquely through the wrist joint. The part by which it remained adherent was composed of a small portion of integument, and tendinous and muscular structure on the radial side of the joint, in which remained probably undivided the superficialis volæ. The radial and ulnar arteries were both divided. Dr. Ames (whose patient he was) placed the parts in apposition, and dressed them in such a way with sutures, adhesive strips, and bandage, that the cut surfaces were kept neatly and accurately in contact. By the second day, however, it was sufficiently evident that union of the parts was impossible, and at Dr. Ames's request I amputated by the double flap operation just above the joint. There was, as Dr. A. informed me, considerable constitutional disturbance; so much indeed as to render the result doubtful for several days; after which the progress of the case was favorable, and the parts united in a reasonable time.

Case V.—October 1st, 1841. I visited, at a distance of ten miles, a negro woman, 28 years old, who four days previously had her hand and fore arm torn and mangled in a most shocking manner, by being caught in a cotton gin. She was under the care of Dr. Roberts, in whose opinion, that amputation was necessary, as there was much swelling even above the elbow joint, and the parts below sloughing rapidly, I entirely concurred. Accordingly I amputated by the double flap operation, immediately above the elbow joint. No unfavorable symptoms supervened after the operation; those previously existing were soon relieved, and in the usual time the parts had healed.

Case VI.—October 28th, 1842. A negro woman, about 35 years of age, subject to epileptic convulsions, during an attack, fell into the fire and burned her hand and wrist so badly that the parts were almost completely charred through.

On the 31st, I visitid her with Dr. McWhorter, and amputated by the double flap operation midway between the wrist and elbow.

She lived twelve miles from town, and Dr. McWhorter did not re-visit her till the third day after the operation, having left directions with the overseer of the plantation, in regard to her treatment. He found her laboring under tetanus, which, from what he could learn, had supervened the day before. She died the day after his visit.

Case VII.—A robust young man, about 21 years old, on the night of the 7th of August, 1843, received a charge of fifteen buck-shot from a gun, which was accidentally discharged while the muzzle was resting in his left axilla and the breech on the floor. The load passed in a mass, grazing the anterior and inferior portion of the humerus, immediately below its head, dividing the artery, veins, nerves, and other soft parts so as completely to denude the bone in that situation. He bled profusely, but the hemorrhage ceased on the supervention of extreme fainting, and the application of some masses of raw cotton to the wound by the bystanders.

Dr. Jones, who resided at a distance of about seven miles, saw him several hours after the accident, at which time he still continued extremely faint. There was neither sensation nor circulation in the arm. As the hemorrhage had not returned, the wound was not disturbed.

On the evening of the 8th, Dr. Smith who resided at a distance of fourteen miles from the patient, saw him; at this time there was incipient gangrene of the arm.

On the night of the 8th, I was requested to visit him, but as he resided some twenty-six miles from Montgomery, I did not see him till 10 o'clock on the morning of the 9th. I found him with a hot dry skin; eyes glassy and brilliant; lips pale and exsanguineous; pulse 120; countenance extremely haggard and anxious. The whole arm was a swollen livid gangrenous mass. The skin covering the shoulder joint above, and the upper portion of the deltoid muscle only had a healthy look and feel; the parts beneath being emphysematous, even a little way above the point at which the appearance of the skin was natural. There was no line of demarcation between the diseased and the healthy parts. We concluded to amputate, as offering a desperate but only chance for life.

A little after 10 o'clock, A.M., he took 60 drops tinct. opii, and a short time before 11 o'clock 30 drops more. At 11 o'clock he was laid on the operating table on his right side, with his head and shoulders a little elevated, and supported with pillows. Dr. Smith compressed the subclavian artery with his thumb, as it passed over the first rib, while Dr. Jones supported the arm. Grasping the mass of muscle and integument covering the shoulder joint with my left hand, I passed a long straight amputating knife through from behind forward, between the acromion process and head of the humerus, cutting out as large a flap as the condition of the parts would admit. As I made the cut, several small arterial branches, near the posterior border of the axilla, threw out blood; and as it was important to economize the vital fluid as much as possible, before proceeding further, I applied ligatures to the two larger, and twisted one or two smaller ones. With a scalpel I next opened the capsular ligament divided the tendons inserted into the tuberosities of the humerus, and raised the head from the glenoid cavity. I now dissected carefully the soft parts from the anterior and axillary aspect of the bone, so as to enable me to grasp with the finger and thumb of my left hand, the mass containing the divided end of the axillary artery. Holding the artery secure in this way, I passed the knife round the head of the bone, and directed its edge downwards so as to divide the posterior border of the axilla, and the ragged remains of the anterior border, separating them clear up to the body, so that their cut edges might be on a level with the parts separated by the shot, and form together a smooth and continuous surface, to be covered by the flap from above. One or two more little arterial branches bled, but were immediately tied; after which I proceeded to apply a ligature to the axillary artery, from which not a drop of blood was lost during the operation. With a pair of strong sharp scissors I cut several ragged projections of integument and muscle, which were in a gangrenous condition, from the lower part of the wound. The flap being drawn down was adapted to the raw surface, which it fitted accurately, by a few sutures and adhesive strips. Over this, lint was applied and secured by several adhesive strips.

He bore the operation with much firmness, making scarcely any complaint. At its conclusion he was much exhausted, his body being covered with perspiration, and his pulse weak, and near 160 in frequency. Frictions with dry mustard and flannel were ordered, and wine ad libitum. This, however, he took with much reluctance.

As it was important that as little blood as possible should be lost, the operation was tedious, as arteries, which, in a patient in a less exhausted condition, would have received no attention, were secured as fast as cut. The time occupied in the operation, dressing the wound, and fixing him comfortably in bed, was about 45 minutes. He lost about six ounces of blood.

He took stimulants of any kind with extreme reluctance, and though for a short time was apparently rallying, died about six hours after the operation.

Case VIII.—A stout, robust, and healthy negro man, about 25 years old, received on the 1st of September, 1843, a heavy charge of bird-shot on the posterior and outer portion of the right leg, midway between the knee and ankle. The muzzle of the gun was so close to him at the time, that the charge was not 'scattered,' but entered as it were in one mass. About an inch and a half above where the shot struck, the fibula was broken, and at the point, a large mass of muscle with its integuments, shot entirely away. About eight days after the accident, the parts having previously swollen very much, mortification commenced in the skin and fascia, at a point between the wound and knee. The swelling of the parts and sloughing continued, and on the 15th I visited him in consultation with Dr. Shoaler, from whom I received the above history.

I found the whole mass of muscle forming the calf of the leg completely denuded of its integuments and fascia, which had sloughed away, and jutting out prominently as if protruded by something lodged beneath. The parts below were swollen and cold, and at one or two points emphysematous. The denuded muscle and cellular tissue, had in some places a sloughy appearance, and the integuments were in the same condition, on the internal surface of the limb, as high up as the inner condyle of the femur. There was much constitutional disturbance and fever, the pulse being 120 in the minute.

He had the character of being a bold, daring, and turbulent fellow, and received his wound while engaged in some rebellious conduct, in which he tried to excite the other slaves of the plantation to join him; nevertheless, at the proposition for operating, he expressed the utmost fear and horror, and actually shewed a disposition to conceal himself to avoid it.

Previous to the operation, he took about half a grain of morphine, and some brandy—of the latter he drank frequently during the time of its performance.

The circulation was arrested with a tourniquet, and the operation performed by making the flaps from the internal and external parts of the thigh, a short distance above the knee. By his struggles the tourniquet became displaced, and as I divided the parts for the internal flap, the blood gushed in a small stream from the popliteal artery; as the vessel was however immediately caught by Dr. Shoaler between his finger and thumb, scarcely any blood was lost. Before proceeding further I applied a ligature. The cellular tissue at the point between the flexor muscles had a suspicious appearance, and was accordingly removed with the scissors.

Though during the operation he lost but little more than a pint of blood, by the time the dressings were applied, he had sunk into a state of the most extreme collapse; being nearly speechless, with a small, quick and weak pulse, and his whole surface bathed in a profuse cold perspiration. By the assiduous use of brandy internally, and frictions with dry mustard externally, he revived.

As he lived at a distance of fifteen miles, I did not see him again, but was informed subsequently by Dr. Shoaler, that the parts sloughed near the place from which the cellular tissue had been removed with the scissors, and that secondary hemorrhage to a considerable extent supervened about the sixth day. He, however, eventually recovered.

Case IX.—Nov. 20th, 1845. I visited a negro man, 30 years old, residing about ten miles from town. Six years previous to my visit a wagon ran over his right leg, midway between the knee and ankle, producing a severe compound comminuted fracture. At the end of three years from the receipt of the injury, he was able to move about a little on the leg; but it was crooked and shortened, immensely swollen, and the soft parts unhealed. Up to the time of my visit he continued to use the leg more or less, but it caused him considerable pain and constitutional disturbance, keeping his system all the time in a febrile state. After going about on it a month or two at a time, he had generally been compelled to take his bed, and remain at rest eight or ten days at least, on account of the extreme suffering thereby produced. The soft parts have never healed.

At present, the leg is swollen to more than twice the natural size. The muscles, cellular tissue, &c., seem agglomerated into a dense mass, having a semi-cartilaginous feel. A small portion of integument and the muscles beneath, posteriorly, just below the knee, have a more healthy appearance. The part, however, is covered with large varicose veins.

There is an ulcer on the inner side of the leg, midway between the knee and ankle, larger than a man's hand, from which exudes a thin, ichorous matter in profusion.

Both the patient and his master were anxious to have the leg amputated, and at as early a period as possible; as they intended soon to remove from the State, and wished to have the parts healed before commencing the journey,

For the purpose of allaying constitutional disturbance and febrile excitement, I confined him to bed, applied emollient poultices, and administered occasionally a small dose of calomel with opium, to be followed by a mild laxative.

As the parties were urgent for the operation, somewhat against my inclination I concluded to perform it on the 29th. There still existed some feverishness of his system, and he had a slight cough, though with the stethoscope no evidences of disease of the thoracic viscera were discoverable. At the earnest solicitations of both patient and master, though contrary to my own judgment, I consented to operate below the knee.

To procure any thing like a sufficiency of even tolerably healthy integument and muscle to form a covering for the stump, I had to operate as near the joint as possible. The knife was passed behind the bones high up, and as much of the parts posteriorly as presented any approach to a healthy appearance formed into a flap. A small flap of integument was also formed anteriorly, and the bones divided immediately below the tibial insertion of the ligamentum patellæ. The artery was divided immediately below its bifurcation, consequently I applied the ligature immediately above. The *venous* hemorrhage was most profuse, in consequence of the varicose condition of the veins, and the unhealthy indurated tissue in which they were lodged, preventing their collapse. In spite of an elevated position of the stump, pressure, &c., they continued to pour forth blood so profusely from their rigid, patulous, and gaping orifices, that to prevent his dying on my hands, I had to apply ligatures to several of them.

Almost exhausted myself, and my two assistants, Mr. McWhorter and Mr. Gindeet, students of medicine, in the same condition, at the end of about an hour and a quarter from the commencent of the operation, the hemorrhage was arrested and the stump dressed.

The patient's pulse, 80 before the operation, at its close was 120, and rather weak, and his skin cool and moist, though he was much less exhausted than I expected he would have been, from the loss of blood, which must have exceeded three pints.

Though the flap was large enough, had it been more healthy, to have covered the stump, yet in consequence of its extreme rigidity, it failed to meet the integuments anteriorly by nearly an inch. Both during and after the operation, I much regretted that I had yielded to the solicitations of the parties to perform it below the knee.

Dec. 2d. Dressed the stump to-day. It has by no means a healthy appearance. The flaps will not meet, and there is a thin serous, unhealthy discharge from the wound. Pulse 110; tongue rough and dry; slight cough. ℞ Hydrarg. subm. ʒss., pulv. opii gr.iii. M. and div. in partes iv æqual. One to be taken every night and morning. Flaps to be held together by moderately drawn adhesive strips, and the stump to be kept enveloped in soft soothing poultices, which are to be frequently changed.

4th. Pulse 96; tongue less dry and rough; appearance of stump slightly improved.

7th. Being unwell myself, Mr. McWhorter, an intelligent and well read pupil, visited him, and dressed the stump, and from his report I judged that he was in rather an improving condition.

On the evening of the 8th he died. All that day he appeared as well as he had been at any time since the operation. Towards evening he complained a little of pain in his breast, made a sudden spring out of bed, and died in less than an hour—speaking no more, but requiring several persons to hold him. The account of the manner in which he died, I received from his master.

Case X.—February 26th, 1844. A large and powerful negro man, 26 years old, was placed under the care of Dr. Baldwin and myself for the purpose of having his leg amputated. His master, who lived at a distance, gave us the following particulars. About eight years ago, in falling a tree, he received an injury on the anterior part of the right leg, a short distance above the ankle joint in front. The bone was not fractured, but several small pieces had at different times exfoliated, and were discharged. The wound never healed, but improved a little once or twice. On the whole, however, the general course has been from bad to worse, and there is now an open ulcer larger than a man's hand, on the anterior and external surface of the leg, above the ankle. There are no sinuses or fistulous openings leading to necrosis or caries of the bone. The whole foot, and the leg commencing about six inches below the knee, are much enlarged, being perhaps twice the size of the sound one, and have an indurated feel, not unlike that of case ix, but in a less exaggerated degree. Always after taking any unusual exercise, the limb becomes more swollen and painful; his system feverish; and he is compelled to keep his bed for several days.

In preparing him for the operation, the parts assumed an improved appearance, and after some entreaty his master gave a reluctant consent to have an effort made to cure the leg without amputating.

By constitutional remedies, the horizontal posture pretty steadily observed, and the use of bandages principally, he was discharged cured sometime in March, 1845. The cicatrix had a tender appearance, and the greater part of it was about the color of the healthy skin of a white man; no deposition of pigmentum nigrum having taken place.

On the 20th of September following his discharge, he was returned to us with his leg in about as bad a condition as when first placed under our care, having become sore soon after his return home.

Oct. 1st. To-day I amputated, a short distance below the knee, forming a large posterior flap of integument and muscle, and a small anterior one of integument. The parts fitted accurately, and in three weeks had healed entirely; the principal part by the first intention. He returned home well in a month after the operation.

Of the ten cases of amputation above related, three, it will be seen, proved fatal, forming a proportion of one in every three and one-third cases. One died of tetanus; another under peculiar circumstances, though from what cause I will not pretend to say, as I did not examine the body after death, and received but an imperfect account of the symptoms at the time from the patient's master. The other fatal case was one in which the most sanguine could have had but a faint hope of success at the time I saw him.

I am satisfied that an aggregate of the cases of amputation from the private practice of a number of physicians, would give a much smaller proportionate mortality than the above, and that any inference drawn from the results in so small a collection would be erroneous. One fatal case in every ten would probably be nearer the truth.

March, 1846.

